# Impact of Active Methodologies Involving Physical Activity on Primary School Students: A Systematic Review (2018–2024)

**DOI:** 10.3390/sports13100358

**Published:** 2025-10-10

**Authors:** Rafael Francisco Caracuel-Cáliz, José Luis Ubago-Jiménez, José Manuel Alonso-Vargas, Eduardo Melguizo-Ibáñez

**Affiliations:** 1 Faculty of Education Science, Universidad Internacional de Valencia (VIU), 46002 Valencia, Spain; 2 Faculty of Education Sciences and Humanities, International University of La Rioja (UNIR), 26006 Logroño, Spain; 3 Department of Didactics Musical, Plastic and Corporal Expression, Faculty of Education Science, University of Granada, 18071 Granada, Spain; alonsojm@ugr.es; 4 Department of Specific Didactics, University of La Laguna, 38200 La Laguna, Spain; emelguiz@ull.edu.es

**Keywords:** holistic development, pedagogical models, physical education, students, well-being

## Abstract

Physical activity integration in elementary education seeks to promote academic performance and the physical, emotional and social health of students. This study aims to examine the effect of active methodologies involving physical activity in primary school students through a detailed review of the scientific literature. A systematic review was conducted regarding PRISMA guidelines. Searches were performed in Web of Science, Scopus and SPORTDiscus. Studies published between 2018 and April 2024 were selected. The studies focused on the application of active methodologies in primary school populations. The quality of the studies was assessed using the Standard Quality Assessment Criteria for Evaluating Primary Research Articles from Various Fields. After screening and review, 22 articles were included. Most of the studies had longitudinal quasi-experimental or repeated measures designs with a randomized cluster-controlled pilot trial. Cross-sectional studies with descriptive data and mixed methods were also included. Cooperative learning and active breaks were found to improve engagement, classroom behavior, and academic outcomes. In addition, gamification and challenge-based learning also showed positive effects on motivation and engagement, although these were more context-dependent. Shorter or small-scale interventions produced promising but less robust results. Active methodologies improve primary education outcomes, but inconsistent designs limit generalization.

## 1. Introduction

Primary education is a key stage in children’s integral development, as it encompasses not only academic learning but also emotional, social, and physical growth [1]. During this period, educators must employ pedagogical strategies that promote the holistic development of students. In this context, active methodologies have emerged as an innovative approach to optimize the teaching- learning process, as they encourage active participation, critical thinking and collaboration, which are fundamental aspects for developing transversal competences in students [2]. Within these methodologies, the incorporation of physical activity plays a central role, since movement-based approaches not only stimulate cognitive engagement but also contribute directly to students’ physical health and socio-emotional development. This is particularly relevant in primary education, where reducing sedentary behavior and integrating structured opportunities for movement can enhance classroom climate, motivation, and academic outcomes. Consequently, the present study focuses specifically on the application of physically active methodologies in primary school contexts, examining how innovative pedagogical strategies that embed movement into the curriculum impact children aged 6 to 12.

Active methodologies, also referred to here as “practice-oriented pedagogical approaches,” are student-centered strategies in which learners actively construct knowledge through hands-on, collaborative, and reflective activities, guided by the teacher [3]. These approaches align with constructivist theory, which conceptualizes learning as an active and contextualized process; socio-cultural theory, which emphasizes the role of social interaction and scaffolding in cognitive development; and self-determination theory, which highlights the importance of autonomy, competence, and relatedness for fostering intrinsic motivation and engagement [4,5,6]. By fostering inquiry, problem-solving, and experiential learning, practice-oriented approaches differ from traditional didactic instruction, positioning students as protagonists in their own learning. These methodologies can be classified into several categories according to the approach they use. For instance, project-based learning encourages students to work on extended projects that require applying knowledge and skills to produce a concrete outcome. Similarly, gamification incorporates the dynamics and mechanics of games into non-playful contexts to foster motivation and engagement in the learning process [7].

It is important to note, however, that the concept of active learning should not be conflated with Physically Active Learning (PAL). While active learning broadly refers to pedagogical approaches that promote student participation and engagement in constructing knowledge, PAL specifically describes the integration of physical activity into academic instruction as a means of enhancing learning and health outcomes. Recent systematic reviews emphasize that PAL is rooted in both educational and movement sciences, and has its own body of evidence showing positive effects on cognitive performance, classroom behavior, and motivation [8,9]. Therefore, in this article, we distinguish between these traditions: active learning as a wider pedagogical paradigm, and PAL as a particular strand that combines curricular content with physical activity.

Research demonstrates that integrating classroom-based physical activity—such as movement-infused lessons—can yield modest yet significant improvements in children’s on-task behavior and academic performance [9]. Moreover, when compared to traditional sedentary classrooms, physically active lessons produced a small positive effect on academic outcomes [8]. Broad meta-analytic evidence further supports that incorporating moderate-to-vigorous physical activity (20–44 min, three times per week) with academic content enhances working memory, fluid intelligence, and math achievement [10,11].

Active methodologies are a set of student-centered pedagogical approaches in which the teacher guides and facilitates the learning process. This type of methodology encourages students’ active participation in the construction of their own knowledge through practical, collaborative, and reflective activities [3]. In contrast to traditional methodologies, where the student assumes a passive role and is the receiver of information, in active methodologies, the student is the main protagonist of learning [12]. These methodologies can be classified into several categories according to the approach they use: cooperative learning, project- or challenge-based learning, gamification, and those that integrate movement and physical activity as part of the educational process [13].

Research has shown the positive impact of active methodologies on different aspects of children’s development, including academic performance, social skills, learning autonomy, and student motivation [14]. Methodologies that integrate movement and physical activity, such as active breaks or dynamic play, have been shown to improve concentration, emotional well-being, and class participation, contributing to a more enriching and healthy educational experience [15,16].

Active methodologies play a fundamental role in promoting physical, cognitive, and emotional development through movement in physical education. This approach recognizes that PE is not only limited to the sporting aspect but also encompasses a broader dimension of holistic student development [17]. Examples of these methodologies include cooperative learning, where students work together to achieve common goals through physical activities; the teaching model of personal and social responsibility, which uses physical activity to foster decision-making, conflict resolution, and self-control skills; and introjective motor practices, which combine physical development with mental well-being through activities such as yoga and dance [12,18].

The implementation of these methodologies in education not only improves academic results but also has a positive impact on students’ physical and mental health. According to Pastor-Vicedo et al. [19], active methodologies that integrate movement can reduce sedentary lifestyles in the school environment, promote healthy habits from an early age, and foster a dynamic and motivating learning environment. In addition, recent research highlights the value of gamification and educational games, which combine playful activities with the teaching of academic concepts, allowing students to learn in a fun and active way [20].

In short, the use of active methodologies, particularly those that incorporate physical activity, offers multiple benefits in the school environment, contributing not only to academic development but also to students’ overall well-being. Recent evidence suggests that these benefits may be explained by mechanisms such as improvements in executive functions (e.g., working memory and inhibitory control), enhanced emotional regulation, and increased classroom engagement and time on task [8,21]. Nevertheless, despite these promising findings, further research is still required to determine how such methodologies can be effectively adapted to diverse educational contexts and student populations, and to disentangle the relative contribution of these mechanisms to learning and development [22]. In this sense, the present review aims not only to synthesize the existing evidence on active methodologies in education but also to critically examine the theoretical and empirical explanations proposed for their impact, thereby identifying current gaps and guiding future lines of research.

Therefore, the aim of this article is to analyze the impact of the use of active methodologies involving physical activity in primary school students through the synthesis and critical analysis of existing studies to identify trends, patterns, and areas of consensus in current research, as well as to highlight limitations and areas of opportunity for future research in this field.

## 2. Materials and Methods

This systematic review was prospectively registered in the International Prospective Register of Systematic Reviews (PROSPERO) international database (ID: 1143791). It examined and analyzed previous research on the impact of active methodologies incorporating physical activity on the cognitive, social, emotional, and physical development of primary school students. The Preferred Reporting Items for Systematic Reviews and Meta-Analyses (PRISMA) statement [23] and the practical guide for systematic reviews, with or without meta-analysis [24], were used to guide the methodological process. The completed PRISMA check-list is provided in the Supplementary File S1.

This work was conducted through a systematic review that examined and analyzed previous research on the impact of active methodologies incorporating physical activity on the cognitive, social, emotional, and physical development of primary school students.

The Preferred Reporting Items for Systematic Reviews and Meta-Analyses (PRISMA) statement [23] and the practical guide for systematic reviews, with or without meta-analysis [24], were used for this purpose.

### 2.1. Inclusion Criteria

The inclusion criteria were as follows:

(a) Time range: Articles published between 2018 and April 2024 were included. This time frame was chosen not only to ensure the inclusion of recent and pertinent studies, but also because bibliometric analyses across databases such as Scopus and Web of Science have shown an exponential increase in research related to physical activity and active learning during this period. For instance, Scopus data reveal that annual publications mentioning ‘physical activity’ surged from approximately 5876 per year in 2001–2017 to around 15,812 per year in 2017–2022—a near three-fold increase—while Web of Science exhibited very similar relative growth.
(b) Availability of the full text of the reviewed studies.
(c) Languages: The articles had to be written in English, Spanish, or Portuguese to cover a broad base of scientific literature in these languages academic field.
(d) Definition of active methodologies related to physical activity: Studies implementing active methodologies specifically linked to physical activity were included. This includes pedagogical interventions that integrate physical movement as part of the teaching- learning process, such as active breaks, learning based on dynamic games, gamification with physical components, or motor activities designed to reinforce academic concepts. While active methodologies cover a wide spectrum of approaches, this study limited the review to those that explicitly incorporate physical activity as a fundamental component.
(e) Target population: Only studies on primary school students were included, in order to specifically analyze the impact of these methodologies at this educational stage.

Study designs were not treated as a separate inclusion criterion; rather, a range of designs—including randomized controlled trials (RCTs), quasi-experimental studies, longitudinal observational studies, and cross-sectional analyses—were purposely considered. This inclusive approach reflects the theoretical relevance of integrating both experimental evidence, which supports causal inference, and ecological insights from observational research to achieve a comprehensive understanding of the impact of physically active methodologies.

The manuscripts were selected according to the following criteria: The bibliographic references of the selected articles were reviewed to identify additional studies that met the same inclusion criteria.

### 2.2. Search Strategy

A systematic review was conducted according to the PRISMA guidelines [23]. Following the Patient/Problem, Intervention, Comparison y Outcome (PICO) strategy [25], the following search formula was used: ((“active method*”) AND (“physical education” OR “phys ed”) AND (elementary school OR primary school OR grade school)). Then, the exploration of articles in three database platforms (Web of Science, Scopus, and SPORTDiscus) was conducted from 1 March to 15 April 2024. This scan was organized into three areas: (1) active methodologies; (2) physical education or physical activity; and (3) intervention, experiment, quasi-experiment, RC trial, or descriptive study. Once the search was completed, duplicates were eliminated.

### 2.3. Study Selection and Processing of Data

**Figure 1:**
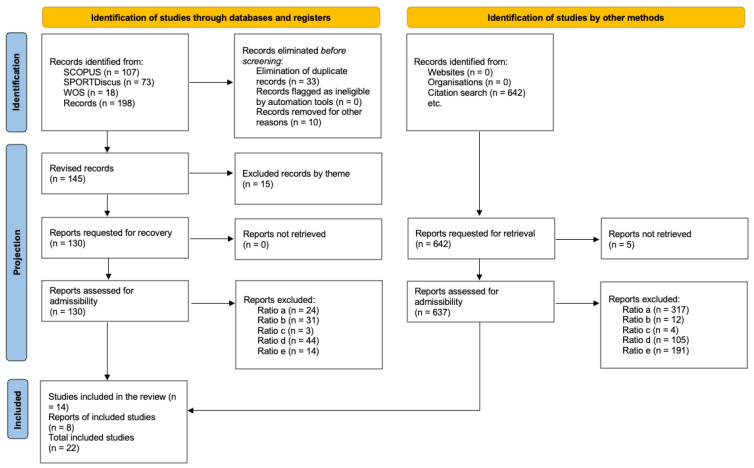
Flowchart.

After completing the article search, both titles and abstracts were reviewed to identify relevant articles and exclude those that did not meet the inclusion criteria. As a result, 22 studies were selected for detailed analysis, focusing on the theme of active methodologies related to physical activity. A top-down search was then conducted by examining the bibliographic references of the included articles, which led to the incorporation of eight additional studies cited in the original papers. Figure 1 shows a flow chart illustrating the process of selecting the articles in the sample.

### 2.4. Quality Assessment

The quality of the selected studies was assessed using a standardized tool, as summarized below (Table 1):

The quality of the articles was assessed using the “Standard Quality Assessment Criteria for Evaluating Primary Research Papers from a Variety of Fields” tool [27]. This tool consists of 14 items addressing aspects such as research design, sample characteristics, methodology employed, data analysis, and results and conclusions presentation. Each item was rated in terms of satisfaction, with values of 2 (satisfactory), 1 (partially satisfactory), 0 (not satisfactory), and 0 (not applicable). The final score was calculated by adding twice the number of satisfactory items plus the value of partially satisfactory items and dividing the result by 28 minus twice the number of non-applicable items. These scores were expressed as percentages ranging from 0% to 100%. Two researchers independently conducted the assessment to ensure objectivity.

### 2.5. Data Collection

In the first phase, data were collected from the selected articles, followed by a thorough verification of the information according to the Preferred Reporting Items for Systematic Reviews and Meta-Analyses guidelines. Particular attention was paid to details related to participants, intervention, comparisons, outcomes, and study design, all of which were in line with the indicated structure. This task was carried out by two experts in the field to ensure consistency in coding and to determine the degree of agreement between researchers in terms of data extraction and selection [28]. A 95% level of agreement was achieved in the classification of articles, which was calculated by multiplying the number of matches by 100 and dividing it by the total number of categories defined for each study, followed by another multiplication by 100. In cases of disagreement, the two researchers discussed the discrepancies and reached a consensus through consultation with a third independent expert, ensuring that the final data selection and coding were accurate and reliable.

## 3. Results

### 3.1. Quality of the Studies

Article quality scores were expressed as percentages, ranging from 0 to 100%, ranging from 0.79 to 1 (Table 2). Inter-rater agreement was calculated using the intra-class correlation coefficient, yielding a score of 0.904 (*p* < 0.001), indicating excellent agreement [20]. After implementing inter-rater agreement, a conservative cut-off point was agreed upon for the selection of raters, including studies with scores of 65% (>0.65). The overall scores ranged from 0.79 to 1 (first observer) and 0.71 to 1 (second observer).

### 3.2. Study Results

To present the main characteristics of the study sample, the data from the articles were coded based on the following units of analysis: (1) Author/s; (2) Country; (3) Context; (4) Subjects; (5) Age; (6) Methodology; (7) Type of study; (8) Duration; and (9) Protocol (Table 3).

For a comprehensive analysis of the results presented in Table 3 and to avoid an over-generalized view of the active methodologies, we can divide the analysis into blocks. This approach will allow for an in-depth analysis of the differences and similarities between the methodologies implemented and the results obtained.

#### 3.2.1. Block 1: Cooperative Methodologies, Gamification and PBL

This block includes studies that have implemented pedagogical strategies that encourage social interaction, cooperation, and Project-Based Learning (PBL). These methodologies were considered movement-based because they intentionally integrate bodily activity into the learning process, either through motor dynamics in cooperative tasks, embodied challenges in gamification, or hands-on, kinesthetic activities in project development. They stand out for their emphasis on students’ active participation and joint work:

- Cañabate et al. (2023) [29];
- Menéndez Santurio (2023) [31]

Nevertheless, short duration and limited samples reduce comparability and limit conclusions on long-term impact.

#### 3.2.2. Block 2: Active Breaks and Didactic Games

This block groups studies that have used active breaks or didactic games as interventions to interrupt sedentary behavior and improve cognitive and physical performance:

- González-Fernández et al. (2023) [30];
- Jiménez-Parra et al. (2022) [33] and Méndez-Giménez et al. (2022) [36];
- Watson et al. (2019) [44]

While most studies reported positive effects, some interventions showed only modest or null changes, particularly when protocols were short or inconsistently applied.

#### 3.2.3. Block 3: Hybrid Models and Innovative Techniques (Flipped Learning, Teaching Personal and Social Responsibility)

This block includes studies that have integrated different active methodologies and innovative pedagogical techniques:

- Botella et al. (2021) [37];
- Muñoz-Parreño et al. (2021) [38];
- Jiménez-Parra et al. (2023) [12]

The heterogeneity of designs makes cross-study comparison difficult, and weaker study designs temper the strength of evidence.

#### 3.2.4. Block 4: Long-Term and Longitudinal Intervention Models

This block groups together studies that applied long-term interventions (more than 6 months), providing a long-term view of the effects of active methodologies:

- Klizienė, Kimantienė et al. (2018) [47] and Klizienė et al. (2018) [48];
- Popa and Popa (2018) [49]

Despite promising outcomes, the limited number of large-scale randomized controlled trials highlights the need for stronger evidence on sustainability.

In summary, the implementation of active methodologies varies significantly in terms of duration, type of intervention, and expected outcomes. Each methodology has a different approach, which makes it difficult to generalize results, as the benefits and impact largely depend on how each approach is applied, the duration of the intervention and the context in which it is developed.

A review of the studies included in Table 3 reveals several clear patterns across countries, duration, and methodology types. Most of the research was conducted in Spain, followed by smaller contributions from Chile, Italy, Romania, Lithuania, and Australia, indicating a concentration of studies in Southern Europe. Participant numbers varied widely, ranging from small samples of 19–25 pupils to larger cohorts exceeding 370 participants, reflecting diverse classroom settings and study scales.

Regarding age, the majority of studies focused on primary school children between 6 and 12 years, with the mean age typically around 9–11 years. Study durations were highly variable, spanning short interventions of 4–6 weeks to longitudinal programs lasting up to 9 months, which may affect both the magnitude and sustainability of observed outcomes.

In terms of methodology, a substantial proportion of the studies employed quasi-experimental or longitudinal designs, often with repeated measures or pretest-posttest structures. A smaller number were pilot randomized controlled trials, cross-sectional descriptive, or observational studies. Most interventions integrated physical activity directly into the academic curriculum, using approaches such as active breaks, movement-based learning, cooperative methodologies, gamification with physical components, and structured motor activities linked to curricular content. Several studies combined traditional teaching with these active methodologies, while others implemented entirely new physical-activity-based approaches.

Overall, these patterns suggest that physically active methodologies are primarily applied in European primary education contexts, with interventions targeting core primary age groups, variable durations, and a range of methodological designs that support both experimental inference and ecological validity. This synthesis complements the detailed information presented in Table 3 and aids in identifying trends, common practices, and potential gaps for future research.

Table 4 reflects a wide variety of active approaches and methodologies applied in the field of PE, with results varying according to each study’s treatment variables and objectives. Each active methodology has particular characteristics that affect its implementation and the obtained results. Following this recommendation, a descriptive and analytical analysis by blocks is presented below.

Block 1: Cooperative and participatory methods

Studies that apply cooperative learning and interdisciplinary programs, such as the Active Values Programme, are notable for their emphasis on social and emotional development. Cañabate et al. [37] pointed out that both cooperative and competitive learning are effective in improving participation and motor performance, with gender differences indicating that boys make more progress in competitive contexts, while the cooperative method is equally effective for both genders. This observation reinforces the idea that the methodology must be adjusted to the characteristics of the group and that the effectiveness of cooperative methodologies is not uniform.

Jiménez-Parra et al. [12] demonstrated that interdisciplinary programs, which combine pedagogical models with active methodologies can contribute to the integral development of pupils, not only physically but also cognitively and socially. This approach underlines the importance of a deeper analysis of interdisciplinarity’s benefits, as its implementation goes beyond the simple use of active techniques: it seeks a holistic impact.

Block 2: Gamification and educational games

As demonstrated in Menéndez-Santurio’s study [31], gamification is a methodology that significantly increases fun, cooperative, and academic learning. These results are not surprising, as gamification is perceived as a more interactive and motivating experience. However, this approach requires further analysis as to how the balance between fun and educational goals can be maintained, as the perception of fun does not always translate directly into better academic outcomes.

Didactic games, used by Yáñez-Sepúlveda et al. [32], have shown a positive impact on the teaching of hygiene habits, highlighting the relevance of games as effective tools for cross-curricular learning. This raises questions about the applicability of didactic games in other more complex areas of the school curriculum and how these methodologies can foster long-term retention of learning.

Block 3: Active breaks

Several studies, such as González-Fernández et al. [30], Jiménez-Parra et al. [33], and Muñoz-Parreño et al. [38], agree that active breaks have positive effects in multiple areas, from improving response time and alertness to increasing physical activity and academic performance. In particular, 10 min breaks are reported to improve efficacy in 10–11 years old schoolchildren and contribute to classroom climate and the reduction in disruptive behavior.

Such interventions, while effective in improving variables such as alertness and motor skills, need to be contextualized. Not all populations or age groups may respond in the same way, so the methodology must be adjusted according to the educational environment. Furthermore, investigating whether the implementation of active breaks has a sustained effect over time or whether its impact is rather temporary would be interesting.

Block 4: Application of responsibility models and active methodologies to the school curriculum

The Personal and Social Responsibility Model applied by Manzano and Valero-Valenzuela [35] and Escartí et al. [45] shows that this methodology, when extended to different subjects, can significantly improve responsibility, autonomy, motivation, and the social climate of the classroom. However, this model requires more structured and consistent implementation to ensure that its benefits are sustained in the long term. Although the results are promising, the success of these programs depends on teacher training and commitment, which raises the challenge of scalability and implementation fidelity.

## 4. Discussion

The results provide a comprehensive view of how various active methodologies implemented in educational settings, specifically in primary education, can influence student development in various aspects. Importantly, physical activity emerges as the central component driving these outcomes, rather than merely a complementary element, underpinning both cognitive and socio-emotional benefits. These findings add to the growing evidence supporting the effectiveness of physical activity-based educational practices.

By comparing the results of these studies with those of previous research that addressed the same variables using different active methodologies, additional conclusions can be drawn about the relative effectiveness of each approach. For example, the implementation of programs such as cooperative learning and active breaks has been shown to improve engagement, motor performance, and learning effectiveness [29,30], results consistent with previous research that has highlighted the benefits of these practices [50,51].

Gamification has emerged as a promising active methodology to improve students’ motivation, engagement, and academic performance [31]. These findings are consistent with those of previous research that found similar benefits of gamification in different educational contexts [52,53]. However, as noted by Domínguez et al. [54] and Sailer and Homner [55], the effectiveness of gamification is highly context-dependent, varying according to game design, duration, and students’ prior experience and receptiveness.

Studies investigating the impact of didactic games and challenge-based learning have shown significant improvements in various skills and knowledge, such as motor skills and cross-cutting learning of hygiene habits [32,49]. These findings support the idea that integrating playful and challenging activities into the school curriculum can improve student participation and engagement while promoting the development of key cognitive and social skills [56].

Finally, results from studies investigating the implementation of the TPSRM have highlighted improvements in student responsibility, autonomy, and motivation, as well as in the classroom social climate [33,45]. These findings are consistent with previous research that has demonstrated the benefits of the TPSR approach in promoting SES and improving school climate [57,58].

It is important to acknowledge the substantial heterogeneity among the included studies, which varied in country, sample size, duration, and type of active methodology applied. Most studies were conducted in Spain, with fewer contributions from Chile, Italy, Romania, Lithuania, and Australia, which limits the generalisability of the findings to other educational contexts. Sample sizes were often small, and intervention durations ranged from a few weeks to several months, potentially influencing the magnitude and sustainability of the observed effects. This variability underscores the need for caution when extrapolating results to different populations and educational systems [8,21]. This underscores the need to interpret results cautiously, particularly in cross-cultural applications.

Potential sources of bias should also be considered. Publication bias may overestimate positive effects, while small sample sizes and the absence of long-term follow-up in many studies limit the strength of the conclusions. Additionally, differences in how active methodologies were implemented—such as variations in intensity, frequency, and fidelity of the intervention—may have influenced outcomes and introduced further variability [9,10].

When situating these findings within the broader international debate on physically active learning (PAL), our results generally align with meta-analyses demonstrating small to moderate improvements in academic performance, executive function, and classroom engagement associated with PAL interventions [8,9,59]. Nonetheless, some discrepancies exist, particularly regarding the long-term impact and the optimal combination of methodologies, highlighting the need for further high-quality, longitudinal research across diverse educational settings.

These findings highlight the importance of using active teaching methods that include physical activity in schools to promote students’ holistic development. Although each active methodology has its advantages and challenges, evidence shows that it can improve academic performance, physical and emotional health, and overall student well-being.

In interpreting these findings, it is also important to consider the methodological and contextual limitations of the included studies. Several interventions received lower quality scores due to small sample sizes, absence of randomization, or lack of long-term follow-up, which limits both statistical power and the robustness of causal inferences. Moreover, the predominance of Spanish and European studies introduces a cultural bias, making it difficult to generalize outcomes internationally. Potential confounding factors—such as socio-economic background, quality of teacher training, and differences in curricular contexts—were rarely controlled for, yet these are likely to moderate the effectiveness of active methodologies. In comparative terms, cooperative learning and active breaks appear more consistently beneficial across outcomes and contexts, whereas gamification and challenge-based learning show greater variability depending on design and implementation. These considerations underscore the need for caution in interpretation and highlight the importance of designing future interventions that are not only methodologically rigorous but also sensitive to cultural and contextual diversity. Taken together, these limitations emphasize the need for balanced interpretations and for future research that systematically addresses these methodological and contextual challenges.

More research is needed to better understand how and why these effects work and to find effective strategies to implement and sustain them in the long term. Future studies should focus on standardizing intervention protocols, exploring long-term outcomes, and including larger, more representative samples to strengthen the evidence base. It is also important to keep in mind the limitations of these studies, such as the difficulty of generalizing results due to differences in how methodologies are applied and possible bias in some research. Future combinations of active methods, the use of new technologies, and the adaptation of approaches for students with special needs should be considered. From an educational point of view, it is essential that teachers receive ongoing training in active methodologies, that these practices are included in curricula, and that teachers and policy makers collaborate to create schools that promote effective and sustainable implementation.

## 5. Conclusions

The studies reviewed show that active methodologies involving physical activity in primary school can have a positive impact on the development of students. In particular, cooperative learning and active breaks stand out as the most consistently supported approaches, with multiple experimental and quasi-experimental studies reporting improvements in physical activity levels, academic performance, and student autonomy. Gamification also shows promise for enhancing motivation and engagement, although its effectiveness appears highly dependent on careful design and implementation. Likewise, the integration of didactic games and challenge-based learning contributes to the development of cognitive and social skills, though the evidence base remains more limited and heterogeneous.

Nevertheless, caution is needed when interpreting these findings. Most studies were conducted in Spain and other European or Latin American countries, which constrains the generalizability of results to broader cultural and educational contexts. Furthermore, the number of large-scale randomized controlled trials remains limited, reducing the strength of causal inferences and the ability to draw firm conclusions about long-term impact.

In conclusion, adopting active approaches to teaching improves not only academic performance but also the overall health and well-being of students, preparing them for future challenges. For educators, this implies the need to systematically integrate movement into classroom routines and curricula, prioritizing evidence-based strategies such as cooperative learning and active breaks. For policymakers, recommendations include embedding physically active learning in educational standards, supporting teacher professional development, and ensuring resources for sustainable implementation. By addressing current methodological gaps and expanding research to diverse contexts, the field can better inform scalable practices that position physical activity as a central driver of holistic student development.

## Figures and Tables

**Table 1:** Summary of the quality assessment procedure for included studies.

Aspect	Summary
Tool	Standard Quality Assessment Criteria for Evaluating Primary Research Papers from a Variety of Fields [26]
Items assessed	14 items covering research design, sample, methodology, data analysis, and reporting of results/conclusions
Scoring	2 = satisfactory, 1 = partially satisfactory, 0 = not satisfactory, NA = not applicable
Final score	(2 × satisfactory + partially satisfactory) ÷ (28 − 2 × NA), expressed as a percentage (0–100%)
Procedure	Two researchers assessed independently to ensure objectivity

**Table 2:** Assessment of the study quality.

Studies	Observer 1	Observer 2
Cañabate et al. (2023) [29]	1	0.95
González-Fernández et al. (2023) [30]	0.95	0.90
Jiménez-Parra et al. (2023) [12]	1	1
Menéndez Santurio (2023) [31]	0.86	0.95
Yáñez-Sepúlveda et al. (2023) [32]	1	1
Jiménez-Parra, Manzano-Sánchez, Camerino et al. (2022) [33]	1	0.95
Jiménez-Parra, Manzano-Sánchez, Camerino, Castañer et al. (2022) [34]	0.79	0.71
Jiménez-Parra et al. (2023) [35]	0.90	0.87
Méndez-Giménez et al. (2022) [36]	0.95	0.90
Botella et al. (2021) [37]	1	1
Muñoz-Parreño et al. (2021) [38]	0.79	0.71
Cañabate et al. (2020) [39]	1	0.95
Muñoz-Parreño et al. (2020) [40]	0.79	0.79
Calella et al. (2019) [41]	0.95	1
Dobrescu (2019) [42]	0.87	1
Manzano & Valero-Valenzuela (2019) [43]	1	0.95
Watson et al. (2019) [44]	0.86	1
Escartí et al. (2018) [45]	0.87	0.79
Méndez-Giménez and Pallasá-Manreca (2018) [46]	0.79	0.86
Klizienė, Kimantienė et al. (2018) [47]	0.86	0.79
Klizienė, Cibulskas et al. (2018) [48]	0.90	1
Popa and Popa (2018) [49]	0.79	0.79

**Table 3:** The main characteristics of the study sample.

Author(s)	Country	Subjects	Age (Mean Age ± Standard Deviation)	Methodology	Type of Study	Duration	Protocol	
							CG	EG
[29]	Spain	92 (55 girls and 37 boys Experimental group = 45 Control group = 47)	9.5 years	Quantitative	Descriptive correlational cross-sectional	8 sessions	Control condition: Competitive methodology	Experimental condition: Cooperative methodology
[30]	Spain	46 (24 girls and 22 boys)	10.75 ± 0.65 years	Quantitative	Descriptive correlational cross-sectional	5 sessions	Control condition: Free reading task	Experimental condition: Active rest (activity based on motor games)
[12]	Spain	102 (Experimental group = 49 Control group = 53)	11.59 ± 0.60 years	Quantitative	Quasi-experimental longitudinal cutting	4 months	Conventional methodology based on direct instruction	Teaching methodology based on the incorporation of physical activity in the classroom within the structure of the Personal and Social Teaching model
[31]	Spain	19 (11 girls and 8 boys)	Between 8 and 9 years old	Qualitative	Cross-sectional descriptive	9 months	NGC	Methodology based on a gamification project called Harry Potter
[32]	Chile	42 (20 girls and 22 boys)	Between 6 and 7 years old	Quantitative	Quasi-experimental longitudinal cutting	4 weeks	NGC	Using educational games in classes Physical Education
[33]	Spain	51 (Experimental group = 26 Control group = 25)	11.73 ± 1.73 years	Quantitative Qualitative	Quasi-experimental longitudinal cutting	3 months	They used strategies based on the imposition of tasks and the establishment of an organization	Application of an Active Break program within the Teaching Model of Personal and Social Responsibility
[34]	Spain	26	11.95 ± 0.63 years	Quantitative Qualitative	Descriptive, inferential cross-sectional	12 weeks	NGC	Three Active Break Methods: Tabata Routines, Active Videos for Physical Involvement, and Active Breaks for Cognitive Involvement
[35]	Spain	250	NR	Quantitative Qualitative	Quasi-experimental with repeated measures (pretest and posttest) of longitudinal section	9 months	Traditional teaching approach, based on the direct instruction teaching technique	Implementation of the educational program, Active Values, based on the adaptation of Active Rest to the methodological structure of Teaching Personal and Social Responsibility in all curricular subjects
[36]	Spain	46 (28 girls and 18 boys Experimental group = 22 Control group = 24)	7.22 ± 0.42 years	Quantitative	Quasi-experimental with repeated measures (pretest and posttest) of longitudinal section	2 weeks	Active Breaks were not introduced	Implementation of the Active Rest program
[37]	Spain	100 (45 girls and 55 boys Experimental group = 51 Control group = 49)	Between 11 and 12 years old	Qualitative Quantitative	Cross-sectional sequential exploratory mixed methods approach	4 sessions	Teaching styles that were already commonly used in the center	Flipped teaching technique Learning through viewing videos created by the principal investigator for this research
[38]	Spain	166 (74 girls and 92 boys Experimental group = 83 Control group = 83)	10.9 ± 0.7 years	Quantitative	Quasi-experimental longitudinal cutting	17 weeks	Active Breaks were not introduced	Received 20 weekly active rest periods combining the physical activity with the curricular content and cooperative learning and emotional intelligence
[39]	Spain	90 (54 girls and 36 boys)	9.5 years	Quantitative	Exploratory cross-sectional approach	6 weeks	NGC	Implementation of the six introjective practices: Yoga, Tai Chi, eutony, active global stretching, Qi -gong and body expression in dance
[40]	Spain	44 (20 girls and 24 boys Experimental group = 22 Control group = 22)	10.44 ± 0.45 years	Quantitative	Quasi-experimental longitudinal cutting	17 weeks	Active Breaks were not introduced	Implementation of Active Breaks
[41]	Italy	47 (23 girls and 24 boys)	8.4 ± 0.3 years	Quantitative	Exploratory longitudinal section approach	3 months	NGC	Conducting two sessions of active breaks in the classroom on three school days a week
[42]	Romania	55 (36 girls and 19 boys)	Between 9 and 10 years old	Qualitative Quantitative	Longitudinal observational section	9 months	NGC	Application of dynamic games based on psychomotor tests
[43]	Spain	25 (11 girls and 14 boys Experimental group = 14 Control group = 11)	9.96 ± 0.84 years	Qualitative Quantitative	Quasi-experimental longitudinal cutting	4 months	Teaching styles that were already commonly used in the center	Application of the program based on the Personal and Social Responsibility Model
[44]	Australia	374 (Experimental group = 138 Control group = 236)	9.22 ± 0.61 years	Qualitative Quantitative	Cluster randomized controlled trial	6 weeks	Active Breaks were not introduced	Three active breaks of moderate intensity and 5 min duration were introduced daily in the classes.
[45]	Spain	170 (87 girls and 83 boys)	9.61 ± 1.2 years	Qualitative Quantitative	Systematic observation	4 months	NGC	Implementation of a school-based TPSR program in physical education and other subject areas
[46]	Spain	199 (64 girls and 55 boys)	10.29 ± 0.97 years	Quantitative	Exploratory longitudinal section approach	9 months	NGC	Implementation of an annual active recreation program
[47]	Lithuania	138 (71 girls and 67 boys) Experimental group = 70 Control group = 68)	Between 6 and 7 years old	Quantitative	Quasi-experimental with repeated measures (pretest and posttest) of longitudinal section	8 months	Teaching styles that were already commonly used in the center	Application of methodology based on the DIDSFA model (dynamic exercise, intense repetition of motor skills, differentiation, reduction in sitting time, etc.)
[48]	Lithuania	98 (51 girls and 47 boys) Experimental group = 50 Control group = 48)	Between 6 and 7 years old	Quantitative	Quasi-experimental with repeated measures (pretest and posttest) of longitudinal section	8 months	Teaching styles that were already commonly used in the center	Application of methodology based on the DIDSFA model (dynamic exercise, intense repetition of motor skills, differentiation, reduction in sitting time, etc.)
[49]	Romania	38 (22 girls and 16 boys Experimental group = 19 Control group = 19)	10.8 years	Quantitative	Quasi-experimental with repeated measures (pretest and posttest) of longitudinal section	5 months	Teaching styles that were already commonly used in the center	Application of movement games through Challenge-Based Learning

**Table 4:** Treatment variables and main outcomes.

Studies	Variables	Active Methodology	Goals	Main Results
[29]	Stake Engine performance Gender	Cooperative learning	To observe gender differences in the efficiency of learning throwing as a fundamental motor skill using two different intervention methods: cooperative and competitive	Both cooperative and competitive learning are effective in improving participation and motor performance, promoting equity in the learning process among students. Gender differences were observed: boys showed greater progress with the competitive approach, while girls and boys achieved similar results with the cooperative method.
[30]	Response time State of alert Effectiveness	Active Breaks	Analyze how Physical Education based on Active Breaks can exponentially impact the school day	Students who took Active Breaks responded faster (365 ms) than those in the Control Condition (379 ms). A student’s alertness changes after a 10 min Active Break (compared to the Control Condition), and Active Breaks improve effectiveness in 10- and 11-year-old students.
[12]	Intrinsic motivation Self-determination Autonomy Index of psychological mediators Responsibility Intention to perform physical activity	Active Values Program	Implement an educational program called Active Values and analyze the psychosocial and cognitive effects of its application	Interdisciplinary educational programs based on the combination of pedagogical models and active methodologies are proposed as methodological alternatives to achieve comprehensive and multilateral development of children and adolescents, as well as to improve the different learning domains of physical education, such as cognitive, social and motor skills.
[31]	Fun Cooperative learning Academic learning	Gamification	To analyze the perception of students, families, and teachers about a Harry Potter gamification project among primary school students.	Significant increase in fun, improved cooperative learning, and increased academic learning thanks to the gamified pedagogical approach
[32]	Hand hygiene Oral hygiene Body hygiene Social hygiene	Educational games	The intervention program in physical education classes, through play, generated significant effects on the transversal learning of hygiene habits.	To determine the effect of using educational games in Physical Education classes on achieving significant cross-curricular learning about hygiene habits.
[33]	Motor skills Postural variations Interrelationships	Active Breaks	Check the results of an active methodology program based on activity breaks	The activity interruption program may be suitable to increase motor participation as well as social and cognitive interaction of students during class.
[34]	Classroom climate Attention	Active Breaks Model for Teaching Personal and Social Responsibility	Verify the results of an active break program within the Teaching Model of Personal and Social Responsibility in the school environment	The results showed an evolution in the behavior of the experimental group from a controlling style to one focused on the transfer of autonomy, while the control group showed an increase in disruptive behavior among students.
[35]	Socio-educational difficulties Sedentary lifestyle Obesity	Active Values Program	Explain the logic and protocol of an educational program called Active Values as an intervention strategy for reducing sedentary lifestyles and promoting values education in schools.	Interdisciplinary educational programs based on active teaching models and methodologies promote the comprehensive and multilateral development of children and adolescents, improving the cognitive, social and motor learning areas in physical education.
[36]	Physical activity	Active breaks	To examine the impact of active breaks on students’ physical activity during classes, recess, and after-school hours, and to explore potential trade-offs during the day.	The results of this study suggest that active break programs that involve both teachers and students in their design could be effective in increasing students’ levels of regular moderate-to-vigorous physical activity. Furthermore, this increase during the school year does not appear to be offset by a reduction in physical activity during the rest of the day.
[37]	Motivation	Flipped Learning	To analyze the effect on student motivation after an intervention with the FL teaching technique, using a Parkour Teaching Unit in Primary Education students	Intrinsic motivation increased significantly, and amotivation scores decreased in the experimental group. The Flipped Learning approach allows for more time in physical education classes and, consequently, is perceived as more fun by students.
[38]	Executive functions Emotional intelligence	Active Breaks	To analyze the changes in executive function variables and emotional intelligence of schoolchildren after the intervention with Active Breaks	The experimental group improved all executive functions that were analyzed, as well as stress control, mood, and the global emotional intelligence index.
[39]	Emotional self-regulation	Introjective Motor Practices	To analyze students’ perceptions of skills and abilities that promote emotional awareness when performing introjective motor practices	The results indicate that after conducting introjective practices in class, a significant improvement of 20.1% was observed in the three dimensions of intrapersonal emotional attention. Furthermore, although there were no significant differences between boys and girls before the test, significant changes were found afterward, with an 8.1% difference in the girls’ results.
[40]	Physical activity	Active Breaks	To analyze changes in students’ physical activity levels after implementing a program based on the Active Break model during the school day.	Following the intervention, there was an increase in total moderate and vigorous activity during physical education classes, other classes, and breaks.
[41]	Physical Activity Sedentary behavior	Active Breaks	To develop and evaluate the feasibility of a classroom intervention that integrates physical activity during school hours and assess its potential effect on reducing inactivity in primary school children.	The program demonstrated a positive effect on reducing inactivity, with a 12 min decrease and a corresponding increase in physical activity levels, including 5 min of moderate/vigorous intensity. Girls were observed to spend less time engaged in light and moderate physical activity, but responded better to the intervention than boys. Both children and teachers expressed high satisfaction with the program.
[42]	Psychomotor skills	Dynamic game	Analyze how movement games used in physical education class can influence in the skills of primary school students	The positive role that movement games play in physical education classes is confirmed if they are used rationally and rigorously selected based on the objectives set, to train the psychomotor skills of primary school children.
[43]	Responsibility Autonomy Motivation Self-concept Social climate of the classroom	Personal and Social Responsibility Model	Apply a program based on the Personal and Social Responsibility Model traditionally used in Physical Education to other educational subjects and evaluate its influence on responsibility, autonomy, motivation, self-concept and social climate in the classroom.	The results indicate improvements in the experimental group’s personal and social responsibility, autonomy, intrinsic and introjected motivation, overall self-concept, and classroom social climate. Consecutively, students’ positive self-evaluations also increased as the program progressed at the various levels.
[44]	Physical Activity Academic Performance	Active Breaks	To conduct a process evaluation to explore factors associated with the feasibility and fidelity of a classroom-based active break program designed to improve student classroom behavior and physical activity outcomes.	Physical activity increased during the intervention’s initial phase but plateaued or declined by the end. Classroom behavior improved immediately after implementing active breaks.
[45]	Physical activity Responsibility	Personal and Social Responsibility Model	To assess the fidelity of implementation of a school-based TPSR program in physical education and other subject areas	The results indicate that the TPSR can provide an effective framework for promoting accountability across the school curriculum.
[46]	Physical activity Fun Intrinsic motivation Satisfaction	Active breaks	To evaluate the annual effect of an active recreation program on fun, intrinsic motivation, satisfaction of basic psychological needs (relatedness, perceived competence and autonomy), intention to practice, possible gender differences and variables predicting intention to practice	Students showed high levels of relatedness, intrinsic motivation, competence, and enjoyment, with no gender differences. Intrinsic motivation and enjoyment predict play during recess and after school. The task-focused program increases motivation and enjoyment, boosting participation in physical activities, especially games, both inside and outside of school. Satisfaction and participation in creating play spaces are important.
[47]	Physical activity Obesity Anxiety	DIDSFA Model	Establish the effects of an intervention program on physical activity and the reduction of anxiety in first grade students	Statistically significant changes were observed in the dependent variables: increased physical activity and decreased obesity. These changes were also observed in the dependent variables: increased physical activity and decreased anxiety in the experimental group.
[48]	Physical activity Physical fitness	DIDSFA Model	To establish the effects of an exercise intervention program on the physical activity and fitness of first-grade students	The exercise intervention program caused statistically significant changes in the dependent variables: increased physical activity and physical fitness for the experimental group.
[49]	Motor skills	Challenge-Based Learning	To verify whether the use of a game program, stages and application paths will improve students’ motor skills.	By using a program that includes games, challenges, and application pathways, an improvement in students’ motor skills was observed. This leads to the conclusion that active participatory methods, such as stages and application pathways, contribute to improving students’ motor skills.

## Data Availability

No new data were created or analyzed in this study.

## Supplementary Materials

The following supporting information can be downloaded at: https://www.mdpi.com/article/10.3390/sports13100358/s1, File S1: PRISMA_2020_checklist.
